# Impact of comorbidities and treatment burden on general well-being among women’s cancer survivors

**DOI:** 10.1186/s41687-020-00264-z

**Published:** 2021-01-07

**Authors:** R. T. Anderson, D. T. Eton, F. T. Camacho, E. M. Kennedy, C. M. Brenin, P. B. DeGuzman, K. F. Carter, T. Guterbock, K. J. Ruddy, W. F. Cohn

**Affiliations:** 1 Department of Public Health Sciences, University of Virginia, PO Box 800717, Charlottesville, VA 22908 USA; 2 Department of Health Sciences Research, Mayo Clinic, Rochester, MN USA; 3 Department of Hematology-Oncology, University of Virginia, Charlottesville, VA USA; 4 School of Nursing, University of Virginia, Charlottesville, VA USA; 5 Carilion Clinic, Roanoke, VA USA; 6 Center for Survey Research, Department of Public Health Sciences and Department of Sociology, University of Virginia, Charlottesville, VA USA; 7 Department of Oncology, Mayo Clinic, Rochester, MN USA

**Keywords:** Treatment burden, Cancer survivorship, Financial insecurity, General health, Comorbidity, Self-management impact, Health literacy

## Abstract

**Background:**

Gains in cancer detection and treatment have meant that more patients are now living with both cancer and other chronic health conditions, which may become burdensome. We used the Patient Experience with Treatment and Self-Management (PETS) framework to study challenges in self-management and its impact on health among survivors of women’s cancers who are caring for other chronic health conditions.

**Methods:**

Applicability of the PETS domains among survivors of women’s cancers with comorbidities was assessed in focus groups to create the study survey. Women surviving primary breast, cervical, ovarian, or endometrial/uterine cancer treated between 6 months and 3 years prior at two large healthcare systems in Virginia were mailed study invitation letters to complete a telephone-based survey. The survey included questions on cancer treatment history, comorbid conditions prior to cancer, treatment and self-management experiences, health literacy, financial security, and items on self-management activities, self-management difficulties and self-management impact (i.e., role/social activity limitations and physical/mental exhaustion). Additionally, general health was assessed with items from the Patient-Reported Outcomes Measurement Information System (PROMIS). Hierarchical regression models and path analysis were used to examine correlates of self-management impact on general physical health (GPH) and mental health (GMH).

**Results:**

Of 1448 patients contacted by mail, 274 (26%) returned an interest form providing their consent to be contacted. Of these, 183 completed the survey. Reasons for non-completion included ineligibility (42), unable to be reached (33) and refusal (6). The majority were survivors of breast (58%) or endometrial/uterine cancer (28%), and 45% resided in non-urban locations. After adjusting for age, race, and cancer type, survivors with higher self-management difficulty reported higher self-management impact, which was associated with lower perceived general health. Reports of higher self-management impact was associated with being single or unmarried, white race, fulltime employed, higher financial insecurity, lower health literacy and more comorbidities. In path analysis, self-management impact was a significant mediator in the association of comorbidity and financial insecurity on GPH and GMH.

**Conclusions:**

Among survivors of women’s cancer, pre-diagnosis comorbidity, health literacy, and financial security are associated with psychosocial impact of self-management and general physical and mental health in the 6 month to 3-year period after cancer treatment has ended. The impact of self-management on psychosocial functioning is an important factor among cancer survivors caring for multiple chronic health conditions. This study provides evidence on the importance of assessing cancer survivors’ self-management difficulties such as in future interventions to promote health and wellness.

**Supplementary Information:**

The online version contains supplementary material available at 10.1186/s41687-020-00264-z.

## Background

Advances in the early detection and treatment of cancer combined with the steady growth in the aging population in the U.S. have resulted in record numbers of cancer survivors who can expect multiple years of survival [1, 2]. The number of people in the U.S. living beyond a cancer diagnosis will increase from approximately 15.5 million survivors in 2016 to 21.9 million by 2029. The concept of “*cancer survivor”* is distinguished as having different phases, such as an acute phase when cancer treatment is still the dominant concern, and extended and lifelong phases when late and long-term effects, and risk for recurrence of cancer are the focus [1, 3, 4].

An aspect of cancer survivorship that has received little attention in the literature is “treatment burden.” This construct refers to a process whereby a patient’s daily workload of self-management, such as the number or type of self-management activities performed (e.g., finding/ understanding information, taking medications, monitoring, maintaining medical appointments), and the challenges or difficulties performing these activities, impacts patient functioning and well-being [5–7]. Cancer survivors may be at particular risk for treatment burden as they manage acute, extended and lifelong effects of cancer on their health and well-being, and strive to prevent recurrence or progression through lifestyle and medical regimens. Factors faced by some cancer survivors such as financial insecurity, low health literacy, and obstacles accessing healthcare or cancer survivorship support services could cause or intensify treatment burden. Cancer patients, as a group, are more likely to be managing other chronic medical conditions compared to persons without cancer [2, 8]. As a result, a new diagnosis of cancer could increase difficulties in ongoing self-management needs such as from medication complexity, additional behavioral or lifestyle restrictions, and the need to access healthcare from multiple providers across different locations or healthcare systems [9].

To better understand the combined challenges of self-management for cancer and chronic health conditions faced by cancer survivors, we conducted a survey study of treatment burden concepts of *self-management activities, difficulties, and their impact* and its relationship to general health among women’s cancer survivors who were managing at least one other health condition at the time of diagnosis. A particular interest in this study was to assess the use of the PETS framework to identify needs of cancer survivors during the extended or lifelong phases, after treatment is completed, and to test the hypothesis that level of self-management challenge is an important mediator of the well-documented association of level of comorbidity and health-related quality of life among cancer survivors [2, 4, 10–14]. This study was conducted under a protocol approved by an Institutional Review Board for health sciences research.

## Methods

### Participant focus groups

Face validity of the PETS treatment burden domains in cancer survivors was assessed using qualitative methods. Focus groups of survivors of women’s cancers who were managing both cancer and a chronic health condition were recruited from multidisciplinary oncology practices at two large healthcare systems to participate in telephone-based group discussion format. Between 4 to 6 patients were recruited per group to call in on a toll-free line at a mutually convenient time. A total of 4 focus groups were held; the groups were led by a trained moderator. Moderated group discussions focused on self-management activities for cancer and concurrent chronic health conditions and were guided by the item domains in the PETS [6]. Participants were asked to consider and describe their major self-management needs and activities (e.g., medication taking, monitoring health, medical appointments, finding health information, exercise, diet, and use of medical devices); difficulties in self-management; and the range of impact of self-management on well-being. Conditions that may influence treatment burden were discussed including financial strain, previous struggles with self-management, and health literacy. Transcripts and meeting notes were prepared and reviewed by the study team and were used to create a brief set of self-management items and to set priorities for inclusion of PETS items and scales in the study questionnaire.

### Survey design and measurement

The final study questionnaire assessed demographic characteristics; aspects of cancer diagnosis, treatment burden concepts of self-management, difficulty, and impact; general health; and selected potential modifiers of treatment burden. Demographic status included, date of birth, county of residence, race, marital status, educational attainment, employment status, income level, and health insurance. Treatment burden items assessed self-management activities and difficulties related to cancer and, separately, for the survivor’s other health conditions. The present study focused on self-management associated with cancer by asking participants whether they did specific self-management activities for their cancer. These items included: taking medications, scheduling medical appointments, monitoring health conditions and behaviors (such as exercise, dieting, body weight, blood pressure, blood sugar), finding reliable health information about cancer, having a routine or program for regular exercise, and needing to use medical devices or equipment for health (such as a glucose monitor, blood pressure cuff or wheelchair). We assessed whether the respondent generally performed each activity for her cancer condition (yes, no, don’t know), and summed the total number as cancer *self-management activities* (see Appendix A). For each activity, we then assessed the level of ease or difficulty (very easy, easy, neither easy nor difficult, difficult, very difficult), which were summed as *self-management difficulties*. We assessed self-management *impact* by asking respondents to rate the extent that their self-management influenced their role and social activities and levels of physical and mental exhaustion. The later were assessed using the PETS impact scales developed by Eton et al. [6]. Perceived general health was assessed using 9 of 10 items from the Patient-Reported Outcomes Measurement Information System (PROMIS) Global-10 scale. These items covered areas of general physical functioning, emotional health, social participation, pain, fatigue, and overall perception of quality of life [15].

Potential modifiers of treatment burden assessed included *number of current comorbid conditions* that require self-management (diabetes, high blood pressure, high cholesterol, depression, anxiety, neuropathy, arthritis, other), a rating of *financial security* using a single item that asked how comfortably participants lived on their current household income (living comfortably on present income/getting by on present income/finding it difficult on present income/finding it very difficult on present income); and *health literacy* using a single item that asked how often participants need help reading instructions, pamphlets, or other written material from their doctor or pharmacy (never, rarely, sometimes, often, always) [16]. *Cancer characteristics* assessed included (cancer type [s], cancer treatment type and end date, year cancer was diagnosed).

### Survey population

All adult survivors of women’s cancers who were within a 6-month to 3-year window from date of last treatment were identified from the institutional patient registries at two large cancer centers in Virginia. Patients treated at one of the participating cancer centers are mostly from small urban areas with a small proportion from rural areas, while the other cancer center serves a mostly rural catchment area. Women were selected for contact if they were age 18 years of age or older, had a diagnosis of breast, cervical, ovarian, or endometrial/uterine cancer, stage I, II, or III, and completed active treatment (surgery, chemotherapy and/or radiotherapy) between 6 months and 3 years prior to the lookup date. To insure that adequate numbers of rural cancer survivors were included from each participating cancer center site despite differences in patient volume and mix, at the larger cancer center which sees mostly urban patients, all cancer survivors with rural or non-metropolitan 5-digit Zip codes were selected for contact (*N* = 411). For the remainder, 50% (*N* = 698) of those living in urban or metropolitan areas were randomly selected for contact. At the smaller cancer center that sees a high proportion of rural patients, all eligible survivors (*N* = 688) were selected for contact. To protect patient privacy, staff at each cancer center mailed an invitation to be contacted and cover letter describing the study to their respective patients. All patients who returned the study invitation card approving contact were attempted for follow-up by telephone. During the telephone call, detailed information was provided about the scope of the study and participation, and treatment window eligibility was self-verified. Survivors who remained eligible and affirmed performing self-management for at least one additional chronic health condition were invited to provide oral informed consent and complete the study survey. The interviews and administration of the surveys were conducted by trained, female interviewers by an academic research center using Computer Assisted Telephone Interviewing (CATI) software to facilitate ease of dialing, track call attempts, and to facilitate data entry including skip patterns and item eligibility.

### Statistical analysis

#### Measures

Cancer self-management difficulty item responses were summed by averaging all self-management tasks respondents reported performing (1-very easy to 5-very difficult). A total score for impact of cancer self-management on psychosocial functioning was calculated by determining separate scores for the two PETS impact scales (i.e. role/social activity limitations and physical/mental exhaustion), transforming the scale scores as 0 and 100, with higher scores indicating greater impact, and taking their mean. For the general health items component scores were derived for physical health (GPH) and mental health (GMH) using PROMIS component score algorithms [15]. Because our physical health measure contained fewer items than the standard PROMIS item set, our GPH score is approximate.

#### Regression and mediation analysis

A model of PETS self-management impact and general health (GPH and GMH) was constructed by testing differences in means for the theoretical covariates using t-tests for regression coefficients and maximum difference in means for multi-categorical predictors using Tukey’s Studentized Range. Covariates examined included age at diagnosis, race, rurality, education, marital status, employment, income, financial security, health literacy, number of comorbidities, cancer type, and chemotherapy. To avoid the risk of over-adjustment, we introduced covariates sequentially according to their hypothesized order of antecedence to self-management impact. Step 1 covariates were age, race, rurality, education, marital status; step 2 added employment and income; step 3 added financial security, and health literacy; step 4 added number of comorbidities, cancer type and chemotherapy, and the final step added self-management difficulties. We expanded this model by including effects of PETS impact score on GPH and GMH. Covariate adjustment was conducted by multivariate regression modeling and tested with the Tukey-Kramer method for multi-category variables. The quantities omega-squared, ω^2^, an estimate of population proportion of variance explained, and partial ω^2^ were used as measures of effect size, and estimate the proportion of variance explained by each predictor, independent of the other predictors considered [17]. Estimates of 0.01, 0.06 and 0.14 have been cited as thresholds for small, medium and large effects [17].

Because comorbidity status has been shown to be strongly associated with cancer survivors’ general health and is also likely associated with self-management difficulty and impact, we hypothesized both direct and indirect pathways could exist in its relationship with general health. Direct effects were considered those that can be attributed solely to level of comorbidity (i.e., assumed to operate through functional impairment or disability), while indirect effects were those operating through self-management difficulty or impact by influencing self-management role functioning and physical/mental exhaustion. A path analysis model was fit to the data using the SAS CALIS procedure, regressing GPH and GMH separately as a function of exposure, covariates, and mediators, and regressing the hypothesized mediators (i.e. self-management difficulties and impact) individually as a function of the covariates. All independent, or, exogenous variables, were allowed to co-vary freely with other exogenous variables. In addition to assuming a linear relationship and approximately normal and symmetrical distribution of residuals, the assumption of no moderation of the mediators on direct effect was tested by conducting statistical interactions, and the assumption of no latent confounding between the exposure-outcome and mediator-outcome was made to identify controlled direct effects (CDE). Decomposition of exposure effects into direct and indirect effects (overall and attributable to each mediator), was made using the ‘EFFPART’ statement in the CALIS procedure.

Missing data were present for income (20 cases, 11% of total) and the self-management difficulty variable (8 cases, 4% of total). When included as covariates in the regression, both variables were imputed using a FCS (Fully Conditional Specification) missing data method available in the SAS system MI procedure (v 9.4), assuming data followed the missing at random (MAR) assumption. For the mediation analysis, instead of imputation, the path model was estimated using Full Information Maximum Likelihood (FIML), which incorporates cases with missing data under the MAR assumption [18].

## Results

**Fig. 1:**
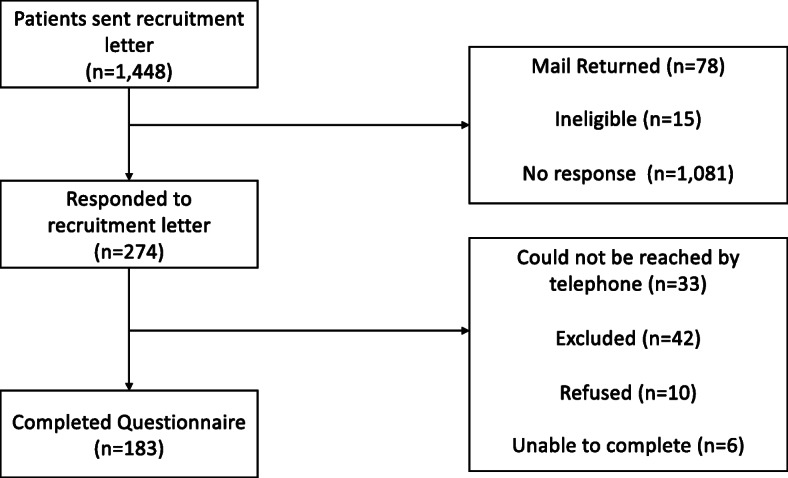
Participant recruitment process

Letters were mailed to 1448 women who were selected based on date of treatment completion for breast cancer (60%), endometrial/uterine cancer (28%), ovarian cancer (7%) or cervical cancer (6%); 86% of patients were listed as non-Hispanic white, and 39% had a last known residence in a non-metropolitan or rural county. The mean age of patients in the sampling frame was 62 years. Figure 1 presents the recruitment and participation rates. After excluding ineligibles and incorrect addresses, a total of 274 (26%) patients returned an interest form, after exhausting contact attempts of at least two weekday telephone calls and one weekend call, 241 respondents were contacted and 199 were confirmed as eligible. Of these, 183 eligible participants completed the telephone survey. Based on American Association for Public Opinion Research (AAPOR) [19], the telephone contact yielded a cooperation rate (i.e. the proportion of successfully contacted households from which an interview is obtained) of 95% and an overall response rate of 81% of those who returned interest forms among those eligible. The total time required to administer the eligibility screen and questionnaire averaged approximately 1 h.

### Study sample

Women cancer survivors recruited for this study had a mean age of 64 years, were mostly white (91%), non-Hispanic (97%), married or living as married (68%) and college graduates (62%) (Table 1). Slightly more than one-third (35%) had an annual household income of greater than or equal to $75,000, while nearly 30% reported an income of less than $35,000. More than half (57%) reported living comfortably on their present household income. Nearly all participants held some form of health insurance (97%), and most (92%) had little need for assistance with health information (i.e., health literacy). Reflecting the sampling design, the sample was largely balanced by urban/rural residential status with slightly more urban residents (53%) than rural (46%), based on rural-urban commuting area (RUCA) county characteristics. Nearly all participants had reported receiving surgery (86%) either alone (34%) or in combination with radiation (23%), chemotherapy (15%), or both (14%) (Table 2). For most participants (92%), the study indexed cancer was their first cancer diagnosis. Although all participants were managing at least one other chronic health condition, approximately 25% reported having no major comorbid illnesses or conditions prior to their cancer diagnosis, while more than one-third (36%) had three or more conditions requiring self-management. The most common concurrent health conditions were high blood pressure (40%), arthritis (39%), hypercholesterolemia (38%), and depression (22%).

**Table 1:** Demographic characteristics

	N	%
Age		
55 years and younger	44	24.0
56–63 years	48	26.2
64–71 years	47	25.7
72–89 years	44	24.0
Total	183	100
Race		
White	167	91.2
Other	16	8.8
Total	183	100
Hispanic ethnicity	5	2.7
Marital status		
Married/living as married	125	68.3
Single or divorced	58	31.6
Total	183	100
Education		
Less than a high school education	15	8.1
High school graduate	32	17
Some college	23	13
2-year college degree	25	14
4- year college degree	33	18
Some graduate work	9	5
Completed professional, masters, PhD, or advanced graduate work	46	25
Total	183	100
Employment status		
Working full-time	51	27.8
Working part-time/not working	120	65.1
Permanently disabled	12	6.5
Total	183	100
Annual household income		
$0 - < $35,000	54	29.5
$35,000 - < $75,000	44	24
$75,000+	65	35.4
Not reported	20	10.9
Total	183	100
Health insurance coverage		
Yes (any kind of coverage)	178	97.5
No	5	2.7
Total	183	100
Health literacy: how frequently need help with written materials from doctor or pharmacy		
Never or rarely	170	92.9
Sometimes	8	4.4
Often or always	5	2.7
Total	183	100
Financial security		
Living comfortably on present income.	104	56.8
Getting by on present income.	49	26.7
Finding it difficult on present income.	16	8.7
Finding it very difficult on present income.	12	6.5
Not reported	2	1.1
Total	183	100
Rural or urban residence		
Urban focused	98	53.5
Rural or urban residence	85	46.4
Total	183	100

**Table 2:** Cancer and comorbid characteristics

	N	%
Cancer type		
Breast cancer	106	57.9
Cervical cancer	9	4.9
Ovarian cancer	12	6.5
Endometrial cancer or cancer of the uterus	51	27.8
Other	5	2.7
Total	183	100
Number of cancer diagnoses		
1	168	92
> 1	15	8
Treatment		
Surgery	62	33.8
Surgery and radiation	43	23.4
Surgery and chemotherapy	28	15.3
Surgery, radiation, and chemotherapy	25	13.6
Other	25	13.6
Total	180	100
Pre-existing conditions		
High Blood Pressure	73	39.8
Arthritis	72	39.3
High cholesterol	69	37.7
Depression	42	*22.2*
Anxiety	34	18.5
Diabetes	27	14.7
Neuropathy	14	7.6
Other pre-existing conditions with fewer than 10 participants (including asthma, hypothyroidism, and osteoporosis)	45	24.5
Number of pre-existing conditions		
0	46	25.1
1	40	21.8
2	31	16.9
3	66	36
Total	183	100

### Self-management tasks and difficulty

Participants self-management activities for cancer survivorship included taking medications (88%), scheduling and attending healthcare appointments (74%), monitoring health (45%), exercise (45%), following a diet plan (31%), maintaining a healthy body weight (32%), using medical devices (32%), and finding needed health information (30%). In Table 3, the overall mean was 4.2 tasks performed out of a possible 8 tasks. The mean difficulty score for these tasks was 1.9 (possible range 1–5) and the mean impact score was 19.1 (possible range 0–100). Self-management tasks performed for cancer general health showed an inverse relationship to impact (more preventive care tasks were associated with less impact). Unlike the mixed results for self-management tasks by category of task, reporting difficulties performing self-management tasks was uniformly associated with higher impact across healthcare categories (results by category not shown).

**Table 3:** Association of self-management tasks and difficulties with PETS impact score

Predictors^b^	β _adj_	Impact Score Effect Estimate (SE)^**a**^
Self-management (all items) # of self-management Items [0–8]	**4.19 (1.50)**	−1.09 (1.10) NS
Cancer specific care	1.60 (1.17)	0.80 (1.42) NS
Other medical conditions care	1.78 (1.70)	3.96 (1.04) ***
General health care related	1.56 (1.28)	−4.85 (1.28) ***
Difficulties (all) Reported self-management difficulty	**1.90 (0.68)**	12.25 (2.27) ***
Cancer care specific	1.80 (0.83)	12.18 (2.24) ***
Other medical condition care	1.79 (0.77)	13.12 (2.60) ***
General health care related	2.07 (0.84)	6.84 (1.94) ***

^a^* *p* < .05, ** *p* < .01, *** *p* < .001. β _adj_ = mean (adjusted) difference using regression weights

^b^Predictors were adjusted for antecedent covariates as follows: age, race, rurality, education, marital status, employment, income, financial security, health literacy

### The importance of comorbidity

In multivariable regression analysis (not shown), each reported comorbid condition present before cancer diagnosis was associated with an increase of 4.2 units in the PETS impact score (*p* < .0041). The mean self-management impact score for number of pre-diagnosis comorbidities of 0, 1, 2 or 3 or more was 10.8, 18.3, 23.5, and 23.4 respectively.

### Predictors of impact

Table 4 shows predictors (*p* < .05) of the PETS impact score and perceived general health (i.e., PROMIS physical and mental component scores). The unadjusted model examining effects for age, race and rurality found no significant predictors (p < .05) of impact. Survivors who were not employed full time or unemployed (**ω**^**2**^ **=** .05), reported financial insecurity (**ω**^**2**^ **=** .10), lower health literacy (**ω**^**2**^ **=** .02), existence of pre-diagnosis comorbidities (**ω**^**2**^ **=** .04), and a higher self-management difficulty (**ω**^**2**^ **=** .13) had a higher psychosocial impact score.

**Table 4:** Predictors of impact and general health status

Unadjusted predictors	Description	Effect measure^b^	Global physical health (GPH)		Global mental health (GMH)		Impact score	
			Effect estimate^**a**^	Effect size (ω^**2**^)	Effect estimate^**a**^	Effect size(ω^**2**^)	Effect estimate^**a**^	Effect size(ω^**2**^)
Age (categorical)	29–55,56-63,64-71,72+	|∆_max_|	3.44	0.00	1.13	0.00	7.37	0.00
Age (continuous)	29–89	β	− 0.04	0.00	0.01	0.00	−0.16	0.00
Race	White vs Others	β	5.54*	0.02	5.49*	0.02	−9.66	0.01
Rurality	Urban Residence vs Rural	β	1.01	0.00	0.86	0.00	0.74	0.00
*Adjusted Predictors*^c^				ω^2^_partial_		ω^2^_partial_		ω^2^_partial_
Education (categorical)	< HS (l), HS-college, Graduate (h)	|∆_max,adj_|	10.82**	0.05	7.52*	0.03	5.51	0.00
Education (ordinal)	1,2,3	β_adj_	4.12**	0.04	3.49**	0.03	−2.35	0.01
Marital status	Married vs Others	β_adj_	3.17	0.01	4.05*	0.03	−7.38	0.02
Employment (categorical)	Disabled (l), < Full Time, Full Time (h)	|∆_max,adj_|	7.63	0.01	5.30	0.02	26.20**	0.05
Income (categorical, non-missing)	0-35 K(l),35-75 K,75 + K (h)	|∆_max,adj_|	6.97*	0.03	6.78*	0.03	4.51	-0.01^d^
Income (ordinal, non-missing)	1,2,3	β_adj_	3.42*	0.03	3.39*	0.03	− 2.28	0.00
Financial security	Comfortable vs Not comfortable	β_adj_	6.55***	0.06	9.71***	0.15	−18.0***	0.10
Health literacy	No help needed vs Help needed	β_adj_	1.81	0.00	3.36	0.01	−8.61*	0.02
No. of prior comorbidities (ordinal)	0,1,2,3+	β _adj_	−3.59***	0.15	−2.35***	0.08	4.17**	0.04
Cancer type (categorical)	Other (l), Endometrial, Breast (h)	|∆_max_|	4.01	0.01	2.90	0.00	2.84	-0.01^d^
Cancer treatment (categorical)	S, RS, CS, RCS, other	|∆_max,adj_|	2.45	−0.01^8^	3.76	0.00	10.48	0.01
Chemotherapy	Yes vs No	β _adj_	−1.25	0.00	−2.70	0.01	4.87	0.00
Self-management difficulty score (continuous, non-missing)	1 = Lowest – 5 = Highest	β _adj_	−0.88	0.00	−1.51	0.01	11.75***	0.13
Impact Score (continuous)	0 = Lowest – 100 = Highest	β _adj_	−0.25***	0.28	−0.22***	0.23	n/a	n/a

^a^ * *p* < .05, ** *p* < .01, *** *p* < .001

^b^|∆_max_| = maximum mean difference, β, β _adj_ = mean (adjusted) difference using regression weights

^c^Estimates were adjusted by differing sets of antecedent covariates chosen a priori for each predictor based on salience and non-overlap from the following list: age, race, rurality, education, marital status employment, income financial security, health literacy, number of comorbidities, cancer type, chemotherapy, challenge, and impact (Please see Appendix B for the specific predictor-covariate sets)

^d^ Although the population parameter $\hat{\omega^2}$estimates is always positive, the estimate can be negative in situations where predictive power is weak

### Predictors of self-rated general health

Also in Table 4, non-white race was associated with lower global physical (GPH) and global mental (GMH) health among the unadjusted predictors. In the adjusted model, having a lower number of comorbidities (**ω**^**2=**^ 0.15), higher financial security (**ω**^**2=**^0.06), higher education level (**ω**^**2=**^0.05), higher income level (**ω**^**2=**^0.03), and white race (**ω**^**2=**^0.02) were associated with higher perceived physical health (GPH). Similarly, higher financial security (**ω**^**2=**^0.15), lower number of comorbidities (**ω**^**2=**^0.08), higher educational attainment (**ω**^**2=**^0.03), higher income level (**ω**^**2=**^0.03), and status of being married (**ω**^**2=**^0.03) were associated with higher perceived mental health (GMH).

### Indirect effects

**Fig. 2:**
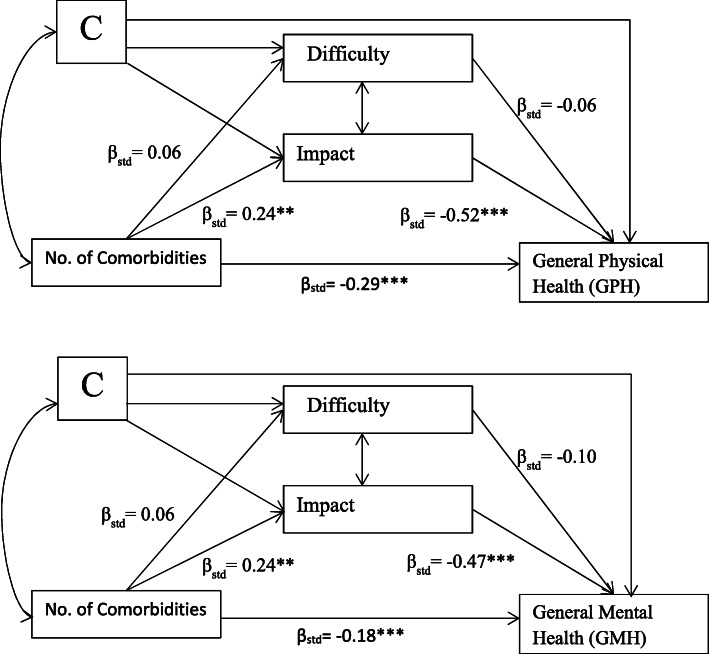
Path Analysis Diagrams. β_std_ represent standardized parameter estimates. Additional predictors (represented as ‘C’) included: age, race, marital status, employment, education, income, financial security, health literacy, cancer type, and chemotherapy treatment. The path model allowed for covariation of any pair of variables without connecting paths

Results for the path analysis that examined the extent that self-management difficulties and impact serve as mediators of the prominent relationship of comorbidity on general health status are shown in Fig. 2. Adjusting for model covariates (i.e., age, race, marital status, employment, education, income, financial security, health literacy, cancer type, and chemotherapy treatment) comorbidity level was found to have both direct effects (GPH: β_std_ = − 0.29, *p* < .001; GMH: β_std_ = − 0.18, p < .001) and significant indirect effects on general health (see Table 5). Increasing the number of comorbidities increases self-management impact, (β_std_ = 0.24, *p* < .01) which in its mediating role, negatively influenced GPH (β_std_ = − 0.52, p < .001) and GMH (β_std_ = − 0.47, p < .001), while self-management difficulty was not found to have a significant mediating role. However, in the path mediation model, when self-management difficulty was allowed to associate with self-management impact it yielded a significant association such that increased difficulty coincided with increased impact. By effect decomposition (Table 5), impact was found to account for most of mediation of the comorbidity - general health status relationship (> 95% of the total indirect effects). Impact was estimated to mediate 30% and 38% of the total effect of comorbidity on GPH and GMH, respectively, and 76% and 40% of the total effect from financial security on GPH and GMH. No statistical interaction between exposures and mediators was found (*p* > 0.05) and therefore interaction terms were not added to the path model. Using the same path analysis framework, other mediation effects on general health for predictors from Table 4 were examined for financial insecurity and health literacy, and are shown in Table 5.

**Table 5:** Decomposition of predictor (direct) and indirect effects on general health

Predictor	Outcome	Total Effect^**a**^	Direct Effect Due to Predictor^**a**^	Indirect Effects^**a**^	Total Proportion Mediated %	Indirect Effect Due to Impact^**b**^	Proportion Mediated By Impact %
# Comorbidities	GPH	−0.416***	−0.286***	− 0.130**	31.3	− 0.127	30.4
# Comorbidities	GMH	−0.301***	−0.181***	− 0.121**	40.2	− 0.115	38.2
Financial Security	GPH	0.261***	0.055	0.206***	78.9	0.198	76.0
Financial Security	GMH	0.439***	0.248**	0.191***	43.5	0.177	40.3
Health Literacy	GPH	0.020	−0.058	0.078**	−^c^	0.068	−^c^
Health Literacy	GMH	0.098	0.019	0.079*	−^c^	0.062	−^c^

Decompositions are over standardized parameter effects

^a^ * *p* < .05, ** *p* < .01, *** *p* < .001

^b^Tests of significance unavailable

^c^Proportion mediated for health literacy was not presented due to the possibility of inconsistent mediation resulting from non-significance of the total effect [20]

## Discussion

This is the first study to comprehensively examine the many facets that comprise treatment burden in cancer survivors and model how they might influence health-related quality of life in the context of other predictors. Our premise was that cancer survivors may be especially vulnerable to burden because not only do they face risk for recurrence requiring lifelong attention, but they may also be managing one or more pre-existing medical condition or have to manage secondary conditions that may arise as a result of cancer treatments [2, 8]. We found that comorbid chronic health conditions were the strongest predictor of physical and mental health scores of the cancer survivors studied, consistent with the literature [2, 10−13]. The results of this study demonstrate that self-management tasks, difficulties and impacts that are integral to the PETS treatment burden framework [6, 9, 21] are inter-related, and that both self-management difficulties and financial insecurity register significant psychosocial impacts among cancer survivors. Self-management impact had a large effect (**ω**^**2=**^0.23, and **ω**^**2=**^0.28) on general physical and mental well-being, with higher self-management impact associated with worse well-being. Number of comorbid conditions had a significant but small effect on self-management impact, but a moderate-to-large effect on general health. Additionally, our analysis showed that the relationship of comorbidity on general health is mediated by level of impact. These findings add to the growing literature on treatment burden for multi-morbidities [9, 22], and raise at least three important points for the study of cancer survivorship. First, risk for negative psychosocial impacts from self-management and lower general health is highest among women with high levels of financial insecurity, who also may have demands from full-time employment and have multiple comorbidities. Second, self-management difficulties, regardless of source or health condition are associated with impact. The latter point could provide support for survivorship programs to broaden their focus by monitoring treatment burden more generally, such as with routine assessments in primary care settings or offer assistance to survivors who find their overall self-management to be burdensome. Third, while self-management tasks for cancer, themselves, do not directly result in psychosocial impact or lower general health, their *combination* with self-management tasks for other ongoing health conditions are associated with negative psychosocial impact and lower self-rated health. One possible explanation for this relationship is the psychological concept of “reserve capacity” suggesting that a patient’s success in coping with new challenges of self-management could depend on the amount of energy and cognitive resources already being expended compared with those held in reserve [23]. Cancer survivors with multiple chronic conditions may become exhausted, leading to role/social activity limitations, and physical/mental exhaustion.

Where our study adds new information to the literature is evidence that the effect of comorbidity on general health cannot be presumed to be entirely direct. We found that approximately one-third of the effect of comorbidity on the general health (30% of physical health and 38% of mental health) among cancer survivors was explained by psychosocial impacts attributed to self-management. Self-management impact also was a strong mediator in the relationship of financial insecurity. Surprisingly, the effect of health literacy on general health is almost entirely mediated by self-management impact. Eton et al. [9] found that self-efficacy predicted general health in cancer survivors after accounting for other variables, but did not find an effect for health literacy.

The results of our study shed light on an important condition of daily life that affect cancer survivors’ health: having self-management difficulties, being single or unmarried which may be a proxy for less assistance with daily self-management, and financial insecurity are associated with an impact from self-management on psychosocial functioning. Although all of the study participants reported self-management for at least one other chronic health condition, more comorbidity was associated with more impact. Future studies are needed to determine if a policy of assessing and managing survivors’ self-management difficulties and impact could result in significant improvement of general health and cancer outcomes of survivors, especially in vulnerable populations.

To our knowledge, this is one of the first studies to have a primary focus on the predictors of treatment burden among survivors of women’s cancers. It is also one of the first descriptive reports on survivor’s self-management activities in the context of co-existing chronic conditions. Self-management activities commonly performed by survivors in this study ranged from information seeking, diet and exercise, to medical treatment and arranging for health care needs. Much of the literature to date on patient need for self-management support has come from work with cardiac patients, especially those with heart failure [24, 25]; and in diabetes management [26], where data provide an evidence base to support developing more comprehensive patient education and support programs [27]. More importantly, cancer survivors, like other mostly older adult patient populations, are likely to have other chronic conditions they are caring for, and therefore, have multiple self-management needs. Cancer may be seen as adding both specific and general challenges, making the totality of self-management important to assess in cancer survivor support programs. .

One of our study objectives was to test whether rural cancer survivors report more indicators of treatment burden than urban survivors who live closer to cancer center treatment facilities. We expected to find greater self-management impact among rural cancer survivors. Our results did not show significant differences in impact by rural/urban residence or geographical distance to the treating cancer center on psychosocial impact or general health. It is possible that rural/urban effects were confounded by poverty status or comorbidity, but more work is needed to understand why longer travel distances to medical care were not more burdensome for the rural cancer survivors in our sample.

Despite using best practices for survey methods [28] and achieving high cooperation rates, our overall participation rate among eligible women was 14%. With a sample size of 182 respondents, our analysis was powered to detect medium to strong effect sizes, which in our estimation, would likely capture relevant clinically significant effects between groups. A related concern is non-response bias [29]. Compared to the sampling frame, the study sample had a similar distribution of cancer diagnoses, but was slightly older with a mean age 64 versus 62 years, and more rural (41% versus 39%). The latter imbalances are likely associated with a slightly higher proportion of white survey respondents (89% versus 86% in the sampling frame). We cannot assess the extent of participation bias along subjective factors such as level of workload difficulties or other patient-reported appraisals. The primary focus of our study was to test a priori hypotheses, motivated by Eton’s PETS framework (2013, 2017) that self-management tasks, difficulties and psychosocial impacts from self-management are interrelated, under the larger concept of treatment burden. We elaborated this framework by positing that self-management impact can mediate effects of comorbidity and other predictors of general health. Our study is valuable by identifying the role that self-management difficulties and impact have in cancer survivors’ psychosocial status. Because so many cancer survivors are simultaneously caring for other chronic health conditions, we argue that a new paradigm is needed in survivorship that considers total treatment burden, not just from cancer, in order to help the individual adjust and thrive as a cancer survivor.

Future studies are needed to examine whether modifying self-management burden can produce improved health status, and whether there are important differences in self-management burden by cancer type. Limitations of our study include the small sample sizes for cancer types other than breast cancer which precluded subgroup comparisons, and that we could only approximate the PROMIS global physical health component score due to the lack of a required item in the scoring algorithm. It is possible that this omitted item resulted in loss of validity and should be considered in comparing the global physical health component score to other research with this measure. Our study focus was exclusively on survivors of women’s cancers based on our prior work and the research interests of our team, our model has not been tested in male cancer survivors who could have different determinants of self-management impact. Gender differences are known to exist in self-management of chronic conditions, and may likewise apply to cancer [30]. Finally, as this was a cross-sectional study we were unable to establish a causal role of comorbidity on general health.

## Conclusion

Among survivors of women’s cancer, being single or unmarried, pre-diagnosis comorbidity, health literacy, and financial security are associated with self-management challenges and difficulties, and lower general physical and mental health in the 6 month to 3-year period following cancer treatment. Negative effects of self-management on psychosocial functioning appear to be a major reason why cancer survivors with multiple chronic health conditions report diminished health-related quality of life. Future studies are needed to examine whether increasing support for cancer survivor’s patient self-management can improve health outcomes, and which cancers are most burdensome to self-manage.

### Supplementary Information

**Additional file 1.** Appendix B Predictor/covariate sets

## Data Availability

The datasets used during the current study are available from the corresponding author on reasonable request.

## Appendix

### Appendix A

Participants indicated yes or no to indicate whether they did each of the following self-management tasks:

- Take medicine, either prescription or non-prescription medicine?
- Have to schedule and keep extra medical appointments for your condition?
- Spend a lot of time finding reliable information about your health condition and how to treat it?
- Have to monitor health behavior and conditions (such as track exercise, watch diet, weigh yourself, check blood pressure, check blood sugar)?
- Have a regular exercise program?
- Follow a specific diet?
- Maintain a healthy body weight?
- Have to use any medical devices or equipment, such as glucose monitor, a blood pressure cuff, or a wheelchair?

## Abbreviations

ACS: American Cancer Society
CDE: Controlled direct effects
FCS: Fully conditional specification
GH: General health
GMH: General mental health
GPH: General physical health
MAR: Missing at random
NCI: National Cancer Institute
PETS: Patient Experience with Treatment and Self-Management
PROMIS: Patient-Reported Outcomes Measurement Information System
RUCA: Rural-urban commuting area
TBI: Treatment burden impact
WHO: World Health Organization
