# Supplementary material for: Impact of comorbidities and treatment burden on general well-being among women’s cancer survivors
Source: J Patient Rep Outcomes. 2021 Jan 7;5:2. doi: 10.1186/s41687-020-00264-z (PMC7790943; doi:10.1186/s41687-020-00264-z)
Supplement: Supplementary file 1 — Additional file 1. Appendix B Predictor/covariate sets [file 41687_2020_264_MOESM1_ESM.docx]

**Appendix B** **Predictor/covariate sets**

| **Predictor** | **Covariates** |
| --- | --- |
| Education (categorical) | age, race, rurality, marital status |
| Education (ordinal) | age, race, rurality, marital status |
| Marital status | age, race, rurality, education |
| Employment (categorical) | age, race, rurality, education, marital status, income |
| Income (categorical, non-missing) | age, race, rurality, education, marital status, employment |
| Income ( ordinal, non-missing) | age, race, rurality, education, marital status, employment |
| Financial security | age, race, rurality, education, marital status, employment, income, health literacy |
| Health literacy | age, race, rurality, education, marital status, employment, income, financial security |
| No. of prior comorbidities (ordinal) | age, race, rurality, education, marital status, employment, income, health literacy, financial security |
| Cancer type (categorical) | age, race, rurality, education, marital status, employment, income, health literacy, financial security, number of comorbidities |
| Cancer treatment (categorical) | age, race, rurality, education, marital status, employment, income, health literacy, financial security, number of comorbidities, cancer type |
| Chemotherapy | age, race, rurality, education, marital status, employment, income, health literacy, financial security, number of comorbidities, cancer type |
| **Mediators** |  |
| Self-care difficulty score  (continuous, non-missing) | age, race, rurality, education, marital status, employment, income, health literacy, financial security, number of comorbidities, cancer type chemotherapy, Burden impact |
| Impact Score (continuous) | age, race, rurality, education, marital status, employment, income, health literacy, financial security, number of comorbidities, cancer type chemotherapy, Self-care difficulty |
